# LINE-1 methylation status and its association with tetralogy of fallot in infants

**DOI:** 10.1186/1755-8794-5-20

**Published:** 2012-06-06

**Authors:** Wei Sheng, Huijun Wang, Xiaojing Ma, Yanyan Qian, Ping Zhang, Yao Wu, Fengyun Zheng, Long Chen, Guoying Huang, Duan Ma

**Affiliations:** 1 Key Laboratory of Molecular Medicine, Ministry of Education, Department of Biochemistry and Molecular Biology, Institute of Biomedical Sciences, Shanghai Medical College, Fudan University, Shanghai, 200032, China; 2 Children Hospital, Fudan University, Shanghai, 201102, China; 3 Department of Forensic Medicine, Shanghai Medical College, Fudan University, Shanghai, 200032, China

**Keywords:** LINE-1 methylation, Tetralogy of fallot, Infants

## Abstract

**Background:**

Methylation levels of long interspersed nucleotide elements (LINE-1) are representative of genome-wide methylation status and play an important role in maintaining genomic stability and gene expression. To derive insight into the association between genome-wide methylation status and tetralogy of fallot (TOF), we compared the methylation status of LINE-1 element between TOF patients and controls. The methylation of the *NKX* 2*–*5, *HAND* 1, and *TBX* 20 promoter regions was also evaluated.

**Methods:**

Genomic DNA from right ventricular tissue samples was obtained from 32 patients with TOF and 15 control subjects. Sequenom MassARRAY platform was performed to examine the methylation levels of LINE-1, *NKX*2-5, *HAND*1 and *TBX*20. Mann–Whitney U test was used to compare differences in methylation levels between two groups.

**Results:**

The methylation level of LINE-1 was significantly lower in patients with TOF, with a median of 57.95% (interquartile range [IQR]: 56.10%–60.04%), as opposed to 59.70% in controls (IQR: 59.00%–61.30%; *P* = 0.0021). The highest LINE-1 methylation level was 61.3%. The risk of TOF increased in subjects with the lowest methylation levels (less than or equal to 59.0%; OR = 14.7, 95% CI: 1.8–117.7, *P* = 0.014) and in those with medium methylation levels (59.0%–61.3%; OR = 2.0, 95% CI: 0.3–14.2, *P* = 0.65). An ROC curve analysis showed a relatively high accuracy of using the LINE-1 methylation level in predicting the presence of TOF (AUC = 0.78, 95% CI: 0.65–0.91; *P* = 0.002). The association of the LINE-1 methylation level with TOF was only observed in males (*P* = 0.006) and not in females (*P* = 0.25). Neither age nor gender was found to be associated with the LINE-1 methylation level in patients or controls. Higher methylation levels of *NKX*2-5 and *HAND*1 and lower methylation levels of *TBX*20 were also observed in patients with TOF than in controls. No association was found between the methylation levels of *NKX*2-5, *HAND*1 and *TBX* 20 with the LINE-1 methylation level.

**Conclusions:**

Lower LINE-1 methylation levels are associated with increased risk of TOF and may provide important clues for the development of TOF.

## Background

Tetralogy of fallot (TOF) is a congenital defect caused by the improper development of the right side of the heart [1]. TOF accounts for 10% of all congenital heart defects, with an incidence of 3.6 per 10,000 live births [2]. It is characterized by four distinct anatomical features: pulmonary outflow tract obstruction, ventricular septal defects (VSD), overriding aortic roots, and right ventricular hypertrophy [3]. TOF malformations can be lethal. Although treatment has advanced dramatically over the past few decades, 0.5% to 6% of TOF patients who survive after treatment suffer sudden cardiac death [4]. Research into congenital heart disease has come a long way since the first description and classification of such conditions. Improvements in utero diagnosis and surgical techniques have considerably brightened the prospects of infants born with congenital heart diseases, but true biological insights into this set of developmental diseases have been gained only recently, and their exact etiology remains unknown [5]. Heredity is likely to play an important role in the development of TOF [6]. In recent years, some studies have proved the existence of a correlation between TOF and gene mutations [4]. *NKX*2-5 and *HAND*1 are known to act as regulatory genes during cardiac development. They are evolutionarily inflexible in their regulation of the differentiation of cardiac muscle cells and morphogenesis of the heart [7,8]. Mutations in *NKX*2-5 and *HAND*1 have been identified in patients with TOF [9]. *TBX*20, a member of the T-box transcription factor family, interacts directly with *NKX*2-5, *GATA*4, and *GATA*5 in the regulation of gene expression in the developing heart [10]. Mutations and over-expression of these genes have been identified in patients with TOF [11]. Some studies have reported chromosomal abnormalities in infants and fetuses with conotruncal cardiac malformations [12]. TOF also has a strong association with San Luis Valley Recombinant Chromosome 8 syndrome and trisomy 21 [13]. The causes of TOF are complex. In addition to disorders in the DNA sequence, epigenetic regulation has been proven to be associated with CHD [14]. Despite advances in uncovering the molecular basis of these epigenetic mechanisms, their roles in cardiovascular development, tissue homeostasis, and cardiovascular disease are largely unknown [15]. Alterations of DNA methylation patterns have been found in many types of cancers, such as lung cancer, brain tumors, and hepatocellular carcinoma (HCC) [16]. The cancer genome is frequently characterized by hypermethylation of specific genes concurrently with an overall decrease in the level of 5′ methyl cytosine. This hypomethylation of the global genome promotes chromosomal instability, translocation, gene disruption, and reactivation of endoparasitic sequences [17].

Long interspersed nucleotide element-1 (LINE-1) is a repetitive element. It constitutes 17–25% of the human genome [18]. LINE-1 elements are moderately CpG rich, and most heavily methylated CpGs are located in the 5′-UTR, where they serve as internal promoters [19]. Because LINE-1 sequences are frequently repeated and widely interspersed human retrotransposons, their methylation level can serve as a surrogate marker of global genomic DNA methylation [20]. Hypomethylation in the promoter region of LINE-1 causes transcriptional activation of LINE-1 element, which causes transposition of the retroelement and chromosomal alteration [21]. One recent report has shown that global LINE-1 hypomethylation can repress genome-wide gene expression [22]. Alterations of LINE-1 methylation status have been observed frequently in some diseases, such as colon cancer [23], neural tube defects [24], and systemic lupus erythematosus [25]. This has been shown to be a good prognostic marker in certain cancers [26]. Maternal LINE-1 DNA hypomethylation has been found to be associated with increased occurrence of non-syndromic CHDs [20]. LINE-1 showing higher methylation levels was also observed in Alzheimer’s disease (AD) [27]. These findings suggest that changes in LINE-1 methylation may not be restricted to cancer but may instead be present in other diseases and show hypo- or hype-methylation status under different conditions.

However, it remains unclear whether changes in LINE-1 methylation are correlated with TOF. Although mutations in *NKX*2-5, *HAND*1 and *TBX*20 have been found in patients with TOF, together they only account for a very small percentage of patients [4]. In addition, little is known about whether changes in methylation are present in these specific genes.

In the present study, to determine whether alterations in LINE-1 methylation exist in the TOF tissue sample and are associated with the risk of TOF, we measured the methylation level of LINE-1 elements in the TOF patients and controls and evaluated the association between LINE-1 methylation status and TOF. The promoter methylation status of *NKX*2-5, *HAND*1 and *TBX*20 and their possible association with LINE-1 methylation were also investigated.

## Methods

### Patients and controls

TOF case subjects were recruited from the Children’s Hospital of the Fudan University, Shanghai, China. Patients were diagnosed by echocardiogram, and the diagnoses were confirmed by surgery. Thirty-two TOF patients undergoing surgical reconstruction were recruited, including 22 (68.8%) male and 10 (31.2%) female patients ranging in age from 1 to 48 months (mean ± SD: 13.4 ± 11.0 months). The control subjects were recruited from autopsy specimens at the forensic medicine department of the Fudan University, Shanghai, China. Fifteen healthy control subjects who had died by traffic accidents were recruited, including 10 (66.7%) males and 5 (33.3%) females ranging in age from 6 months to 37 years (mean ± SD: 19.8 ± 13.9 years). All the tissue samples obtained from right ventricular outflow tracts were saved in RNAlater® ( AMBION, Inc., Austin, USA) immediately after surgical resection or autopsy and stored until use.

This study was approved by the local ethics committee of the Fudan University. Written informed consent was obtained from the parents and relatives of all study subjects. Clinical features of the study subjects are summarized in Table 1.

**Table 1:** Clinical characteristics of study subjects

**Characteristic**	T**OF (n = 32)**	**Control (n = 15)**
Age (mean ± SD)	19.8 ± 13.9 (months)	13.4 ± 11.0 (years)
Male (%)	22 (68.7)	10 (66.7)
Female (%)	10 (31.3)	5 (33.3)

### DNA extraction and sodium bisulfite conversion

Genomic DNA was extracted from the heart tissue samples using a QIA amp DNA Mini Kit according to manufacturer’s instructions (Qiagen, Hilden, Germany). The concentration and purity of the DNA were determined by absorbance at 260 and 280 nm by NanoDropTM 1000 Spectrophotometer (Thermo Scientific, Wilmington, USA). Sodium bisulfite modification for the extracted DNA was performed using an EZ DNA Methylation Kit™ strictly according to manufacturer’s instructions (Zymo Research, Orange, CA, USA). Sequencing results confirmed that more than 99.0% of cytosine residues were converted. The bisulfite-converted DNA was re-suspended in 10 μl elution buffer and stored at −80 °C until the samples were ready for analysis.

### Quantitative MassARRAY analysis of gene methylation status

**Figure 1:**
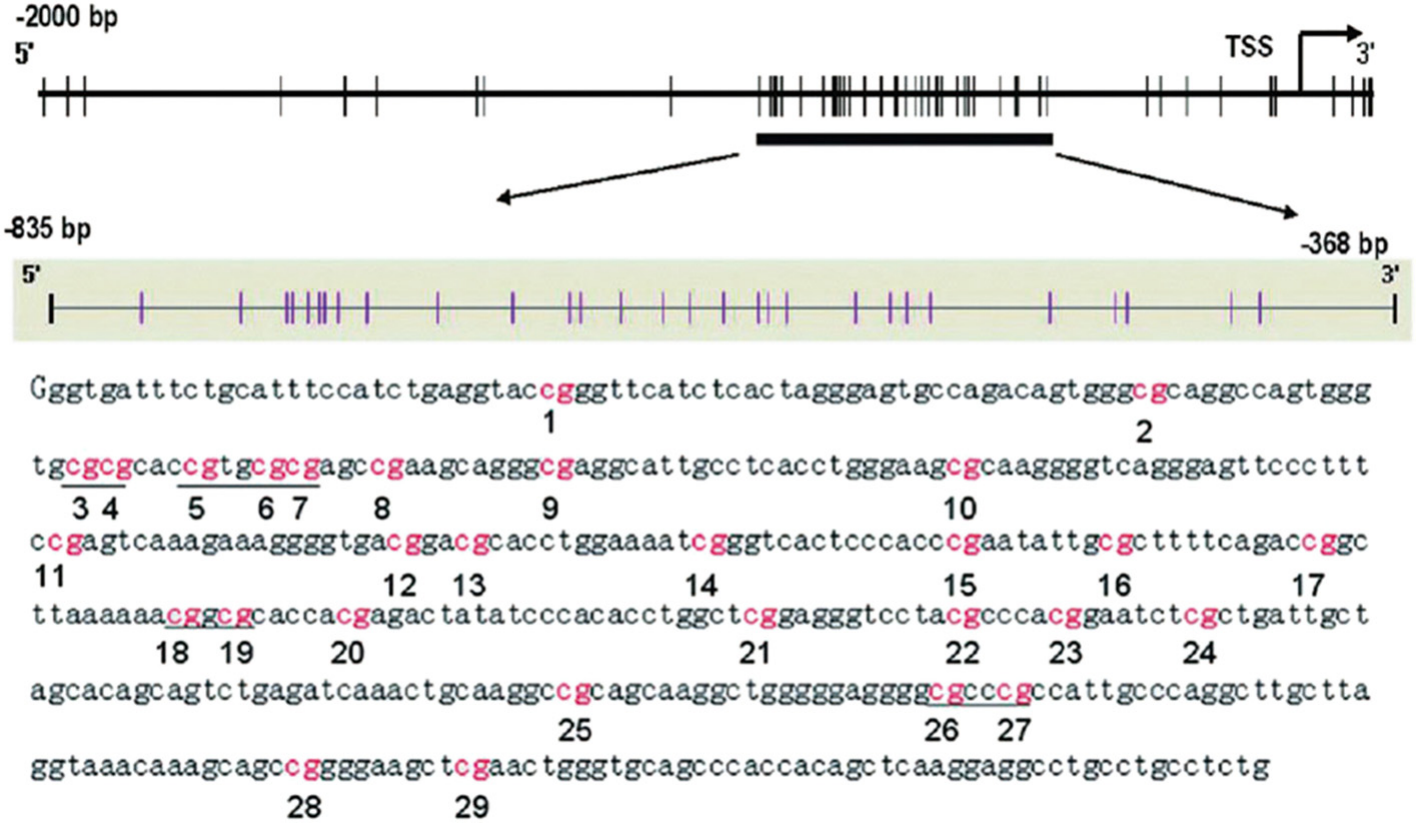
**Long interspersed nucleotide element-1 (LINE-1).** The sequence shown represents a 468 base pair fragment (positions ^-^835–^-^386) in the 5′-UTR of LINE-1. Numbers 1–29 refer to locations of the CpG site within the LINE-1 elements tested, and the underlining highlights the CpG units that include more than one CpG site tested; TSS, transcription star sites

The Sequenom MassARRAY platform was used to perform the quantitative methylation analysis of LINE-1 element. This system, which combines base-specific enzymatic cleavage with MALDI-TOF mass spectrometry, is a highly accurate, sensitive and high-throughput method for the quantitative analysis of DNA methylation at CpG sites [28]. The robustness of this approach for quantifying methylated and unmethylated DNA has been demonstrated by the Sequenom groups [29]. The region analyzed and the CpG sites of LINE-1 promoter are shown in Figure 1. Moreover, the same method was also used for analysis of the promoter methylation status of *NKX* 2–5, *HAND*1 and *TBX*20. The primers used in this study were designed using Methprimer (http://epidesigner.com; Table 2). For each reverse primer, an additional T7 promoter tag was added for in vivo transcription, and a 10-mer tag was added to the forward primer to adjust for the melting temperature differences. Briefly, the 5 μl PCR mixture contained 10 ng bisulfite-treated DNA, 25 mM dNTP, 0.2 U of Hot Start TaqDNA polymerase (Sequenom, Sequenom Inc., San Diego, CA, U.S.), and a 1 μM mixture of forward and reverse primers. The PCR mixture was pre-heated for 4 min at 95 °C and then incubated for 45 cycles of 95 °C for 20 s, 56 °C for 30 s, and 72 °C for 60 s, followed by 72 °C for 3 min. Two microliters of SAP mix containing 1.7 μlH_2_O and 0.3 μl (1.7 U) of shrimp alkaline phosphatase (Sequenom) was added to digest redundant dNTPs with the following program: 37 °C for 20 min, 85 °C for 5 min, then maintained at 4 °C.

**Table 2:** Primer sequences, position, product length, and CpG units used for MassArray quantitative methylation analysis

**Genes**	**Forward primer (5′ → 3′)1**	**Reverse primer (5′ → 3′)2**	**Position**	**Product length (bp)**	**CpG unit**
LINE-1	TTTTATTAGGGAGTGTTAGATAGTGGG	CCCCAAAAATAAAACCTACAAAAAC	−835–-368	468	24
NKX2-5	AGGAGGGTTTTGGATTTTTTTT	ATTTATTCCCAAACCTCTACTCCTC	−59–426	486	21
HAND1	GAGGAGATTTGTTGGTTAGATGTTT	AATAAAAATTCCAACAATTCCCAAT	−886–-414	473	25
TBX20	TTTGAGTGTGTATGTTAGTTTGAGTTT	CTCCTATTTTCCCTAAAAAAACCCT	−945–-635	311	17

^1^10-mer tag: cagtaatacgactcactatagggagaagg and ^2^ T7 promoter tag: aggaagagag were added.

Five microliters of T Cleavage Transcription/RNase Cocktail, including 0.89 μl 5x T7 polymerase buffer, 0.24 μl T cleavage mix, 3.14 mM dithiothreitol (DDT), 22 U of T7 RNA and DNA Polymerase, 0.09 mg/ml RNase A, and 2 μl of product of the PCR/SAP reactions were mixed and incubated under the following conditions: 37 °C for 3 hours of in vitro transcription and RNase A digestion. Then the mixture was further diluted with H_2_O to 27 μl, purified with CLEAN resin (Sequenom) and robotically dispensed onto silicon chips preloaded with matrix (SpectroCHIP; Sequenom). The spectra and the methylation values of matrix-associated laser desorption/ionization time-of-flight mass spectrometry (Sequenom) were collected and analyzed using Epityper software (version 1.0; Sequenom).

All experiments were performed in triplicate. Inapplicable readings and their corresponding sites were eliminated from analysis. The methylation level was expressed as the percentage of methylated cytosines over the total number of methylated and unmethylated cytosines.

### Statistical analysis

Data were analyzed using GraphPad Prism (version 5.0; GraphPad Software Inc., San Diego, CA, U.S.) and SPSS (version 13.0; SPSS Inc., Chicago, IL, U.S.). Mann–Whitney U test was performed to compare the methylation levels between the TOF and control groups and between male and female subjects. The methylation levels were classified as quartiles according to their distributions in controls, and the highest quartile was used as the reference group for risk estimation. Odds ratios (ORs) and 95% confidence intervals (95% CI) were calculated to estimate the risk of TOF in different methylation levels using logistic regression. To further explore whether the hypomethylation of the LINE-1 element could serve as a prognostic indicator for incidence of TOF, receiver operator characteristic (ROC) curve analysis was performed to determine the accuracy in predicting the presence of TOF. The area under the curve (AUC) was calculated to evaluate the discriminatory capacity [30]. T-test was used to compare differences in age between the TOF and control groups. Chi-square test was used to compare the differences in gender between the two groups. Spearman correlation analysis was performed to evaluate the correlations between the methylation level of LINE-1 and the methylation levels of *NKX*2-5, *HAND*1 and *TBX*20 and age. All statistical analyses were 2-sided and *P* < 0.05 was considered statistically significant.

## Results

### LINE-1 methylation levels in the TOF patients and controls

**Figure 2:**
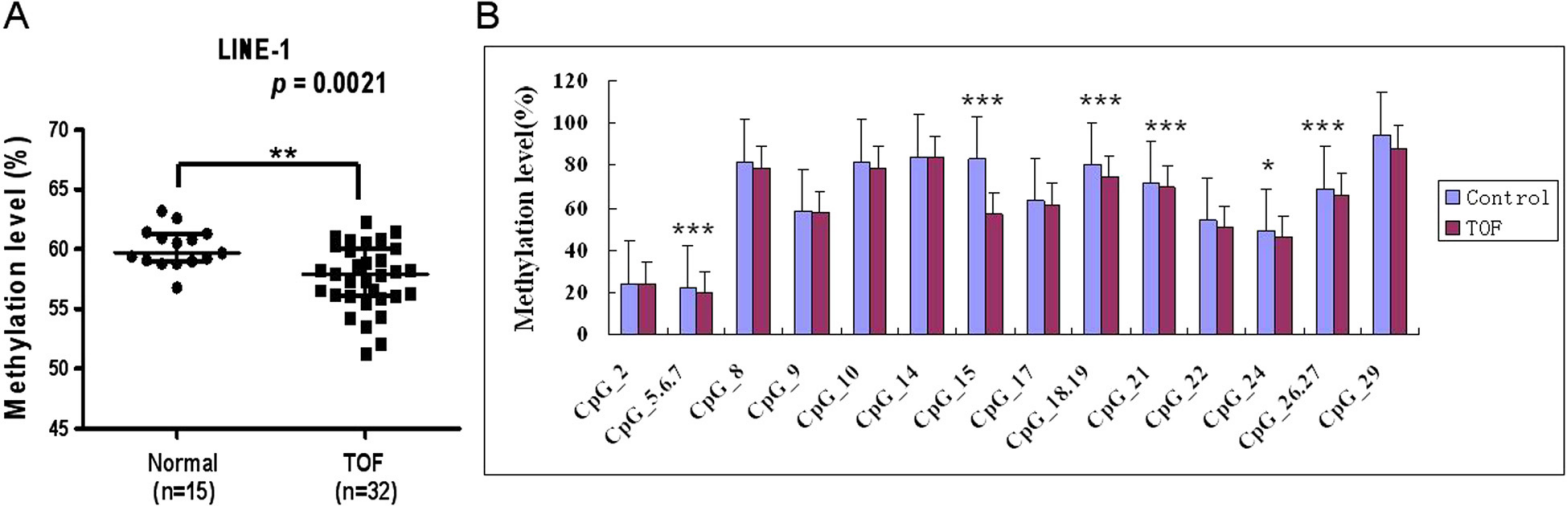
**(A) Median methylation levels of long interspersed nucleotide element-1 (LINE1) between control and TOF subjects. (B)** Median methylation levels of 14 informative CpG units in LINE-1 between control and TOF subjects. Control, n = 15; TOF, n = 32. *P <0.05, **P < 0.01, ***P < 0.001 (Mann–Whitney U test)

To determine the whole-genome methylation level, we analyzed the methylation status of the LINE-1 element in 32 patients with TOF and 15 control subjects. To exclude any tissue heterogeneity that might affect methylation levels, we used tissue taken from similar regions of the right ventricular. The amplicon detected in the 5′-UTR of LINE-1 was 468 base pairs in length and contained 29 CpG sites which could be divided into 24 CpG units. Prior to analysis, strict quality control was carried out to remove potentially unreliable measurements, such as low mass, high mass and silent peak overlap CpG units. The CpG units that failed to produce data for more than 30% of samples (unreliable CpG units) and samples lacking more than 30% of their data points (unreliable samples) were discarded [31]. The methylation level of LINE-1 was significantly lower in patients with TOF, with a median value of 57.95% (interquartile range [IQR]: 56.10%–60.04%), as opposed to 59.70% (IQR: 59.00%–61.30%) in controls (*P* = 0.0021, Figure 2A).

The methylation level of every CpG unit was also evaluated. After the removal of unreliable data, we obtained 14 informative CpG units containing 18 CpG sites. The mean methylation levels varied across different CpG units, ranging from 19.8% to 94.3% (Figure 2B). The methylation levels at CpG_5.6.7, CpG_15, CpG_18.19, CpG_21, CpG_24, and CpG_26.27 were significantly lower in patients with TOF than in the controls (*P* < 0.05). No significant differences were found at the other CpG units (*P* > 0.05).

### Association between LINE-1 methylation and the risk of developing TOF

Twenty-two patients with TOF (68.7%) were grouped into the lowest quartile (methylation level less than or equal to 59.0%). Only 2 TOF patients (6.3%) were grouped into the highest quartile (methylation level greater than or equal to 61.3%). Eight TOF patients (25.0%) were categorized into the medium quartile (methylation level between 59.0% and 61.3%). Subjects in the lowest quartile had a greater risk of TOF than those in the highest quartile (OR = 14.7, 95% CI: 1.8–117.7, *P* = 0.014). Subjects in the lowest quartile were not found to have significantly greater risk of TOF than those in the medium quartile (OR = 2.0, 95% CI: 0.3–14.2, *P* = 0.65; Table 3).

**Table 3:** Association between LINE-1 methylation levels and risk of TOF

**LINE-1 methylation level**	**TOF (%)**	**Control (%)**	**OR (95% CI)**	***P***
	**(n = 32)**	**(n = 15)**		
Highest quartile (>75%)	2 (6.3)	4 (26.7)	1.0 (reference)	
Medium quartile (25%–75%)	8 (25.0)	8 (53.3)	2.0 (0.3–14.2)	0.65
Lowest quartile (<25%)	22 (68.7)	3 (20.0)	14.7 (1.8–117.7)	0.014

*OR*, odds ratio; *P*, percentile; Cutoffs defined as 25% P and 75% P of the control group methylation level.

**Figure 3:**
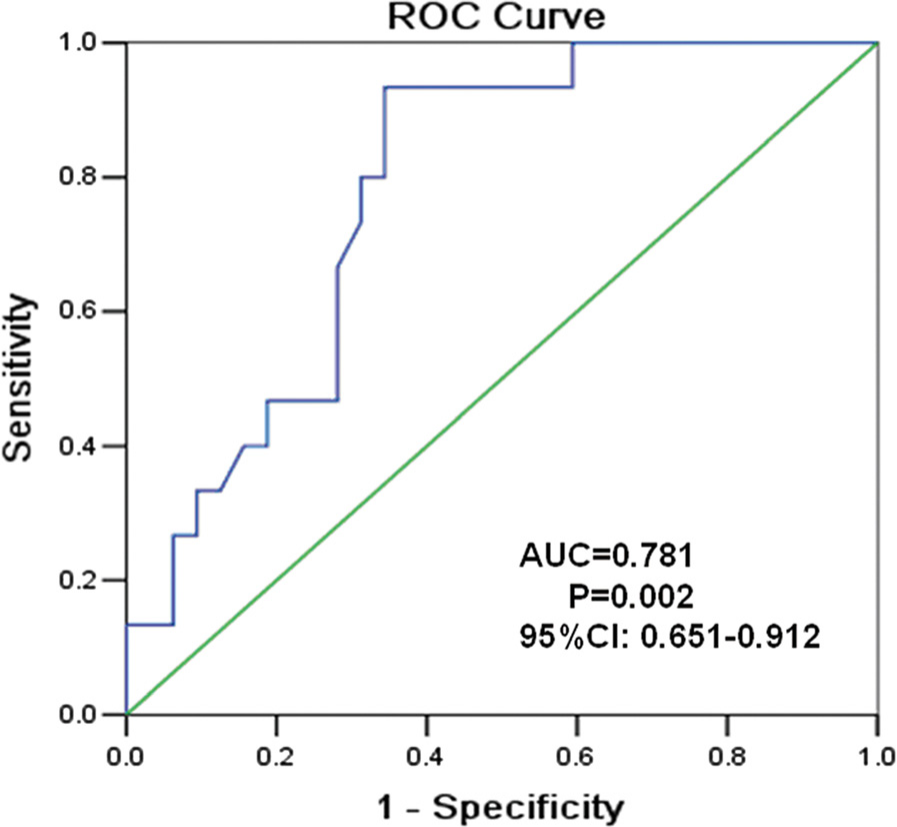
Receiver-operator characteristic (ROC) curve indicating the discriminatory accuracy in predicting the presence of TOF by LINE-1 methylation level.

ROC curve analysis revealed that the AUC value for the LINE-1 methylation level was significantly higher in the TOF patients (AUR = 0.781 for controls *vs.* TOF patients, 95%CI: 0.65–0.91, *P* = 0.002; Figure 3).

### Association between of LINE-1methylation and age

**Figure 4:**
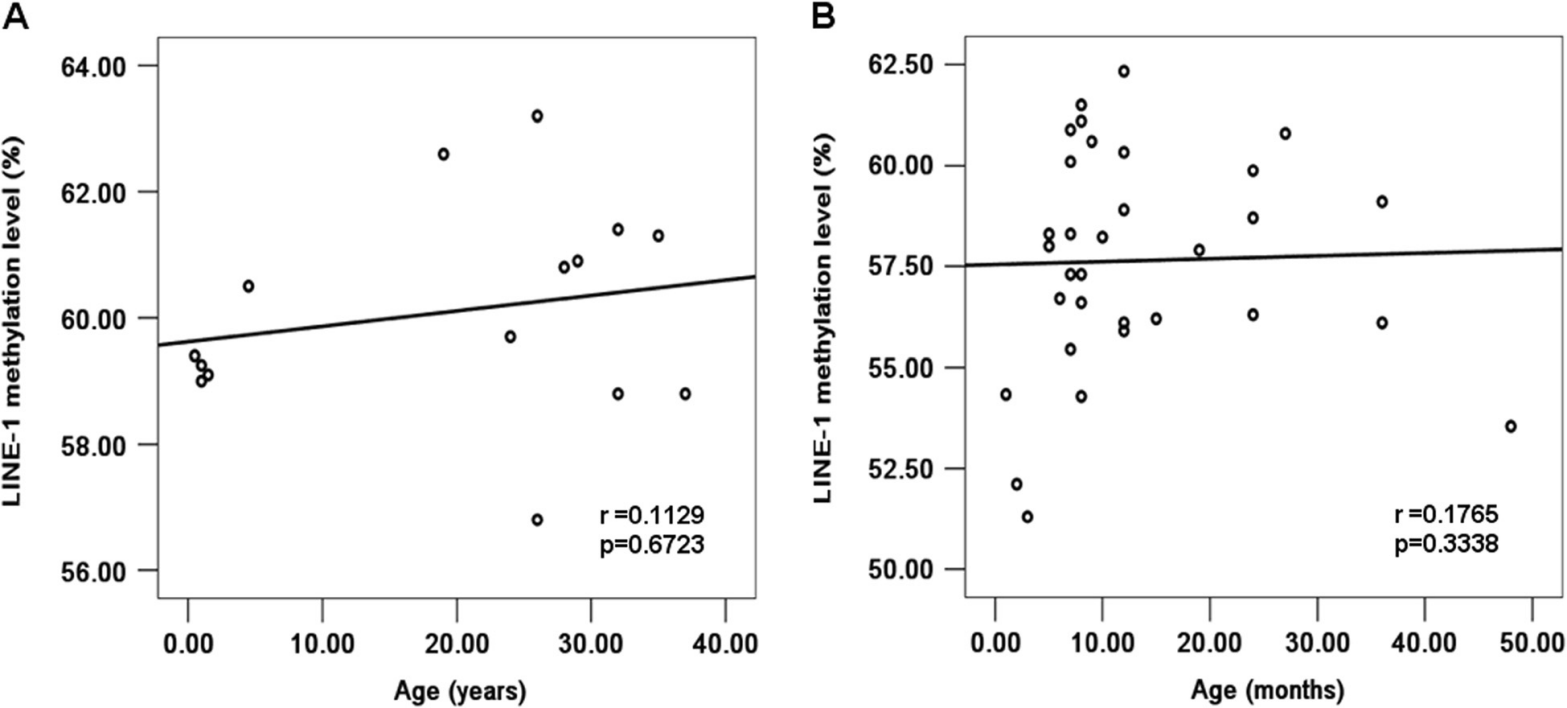
(A) Correlation between LINE-1 methylation level and age in the control group (n = 15) and in the TOF patients (n = 32).

No correlation was found between the LINE-1 methylation and age in the control group (r = 0.11, *P* = 0.67; Figure 4A) or in TOF patients(r = 0.18, *P* = 0.33; Figure 4B).

### Association between of the LINE-1 methylation and gender

**Figure 5:**
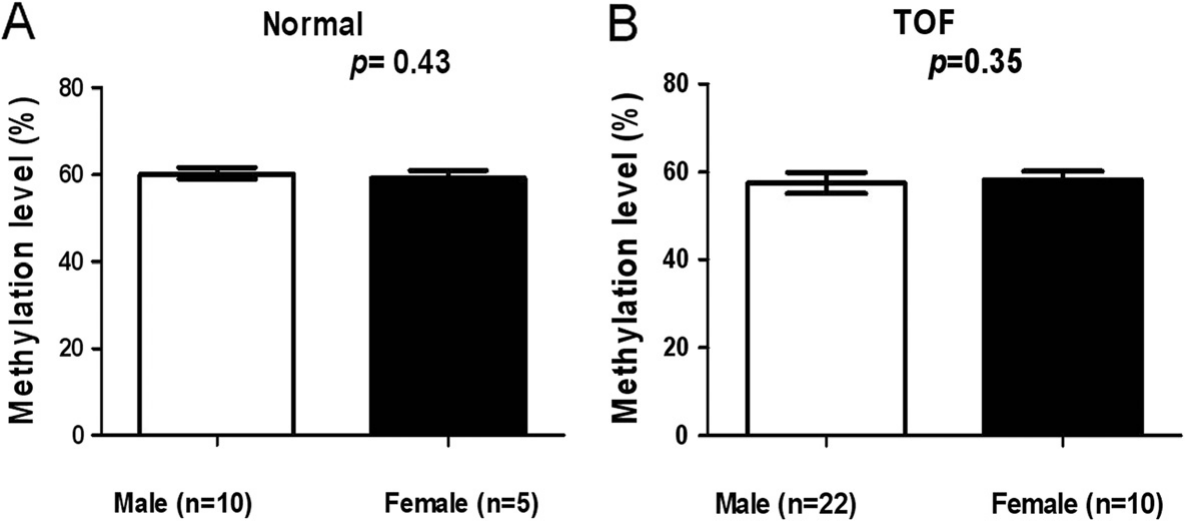
**Association between LINE-1 methylation level and gender in (A) control subjects and (B) TOF patients.** *P < 0.05, **P < 0.01, ***P < 0.001 (Mann–Whitney U test).

**Figure 6:**
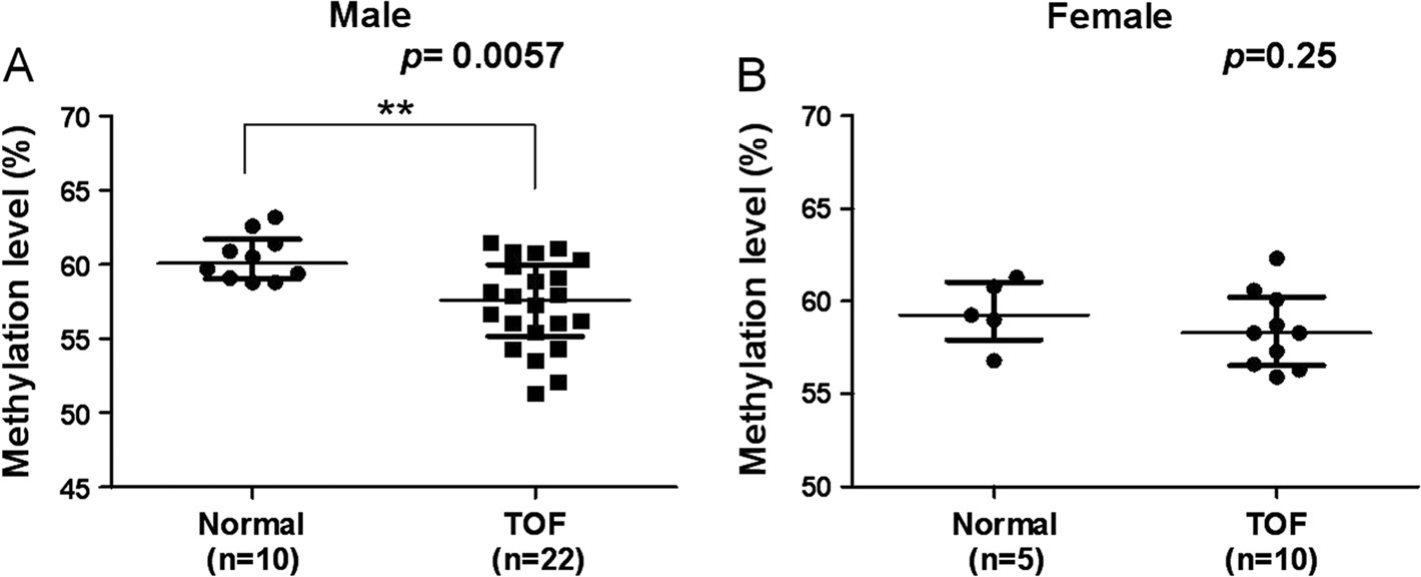
**Association between LINE-1 methylation level and TOF in (A) males and (B) females.** *P < 0.05, **P < 0.01, ***P < 0.001 (Mann–Whitney U test)

No significant difference was found between the median LINE-1 methylation levels of male and female control subjects (60.1% *vs.* 59.3%, *P* = 0.43; Figure 5A) or in TOF patients (57.6% *vs.*58.3%; *P* = 0.35; Figure 5B). Male TOF patients had significantly higher median methylation levels than male controls (60.1% *vs.*57.6%, *P* = 0.0057; Figure 6A). There was no significant difference in the median LINE-1 methylation level among female subjects (59.3% *vs.* 58.3%, *P* = 0.25; Figure 6B).

### Association between LINE-1 hypomethylation and NKX2-5, HAND1, and TBX20 methylation levels

**Figure 7:**
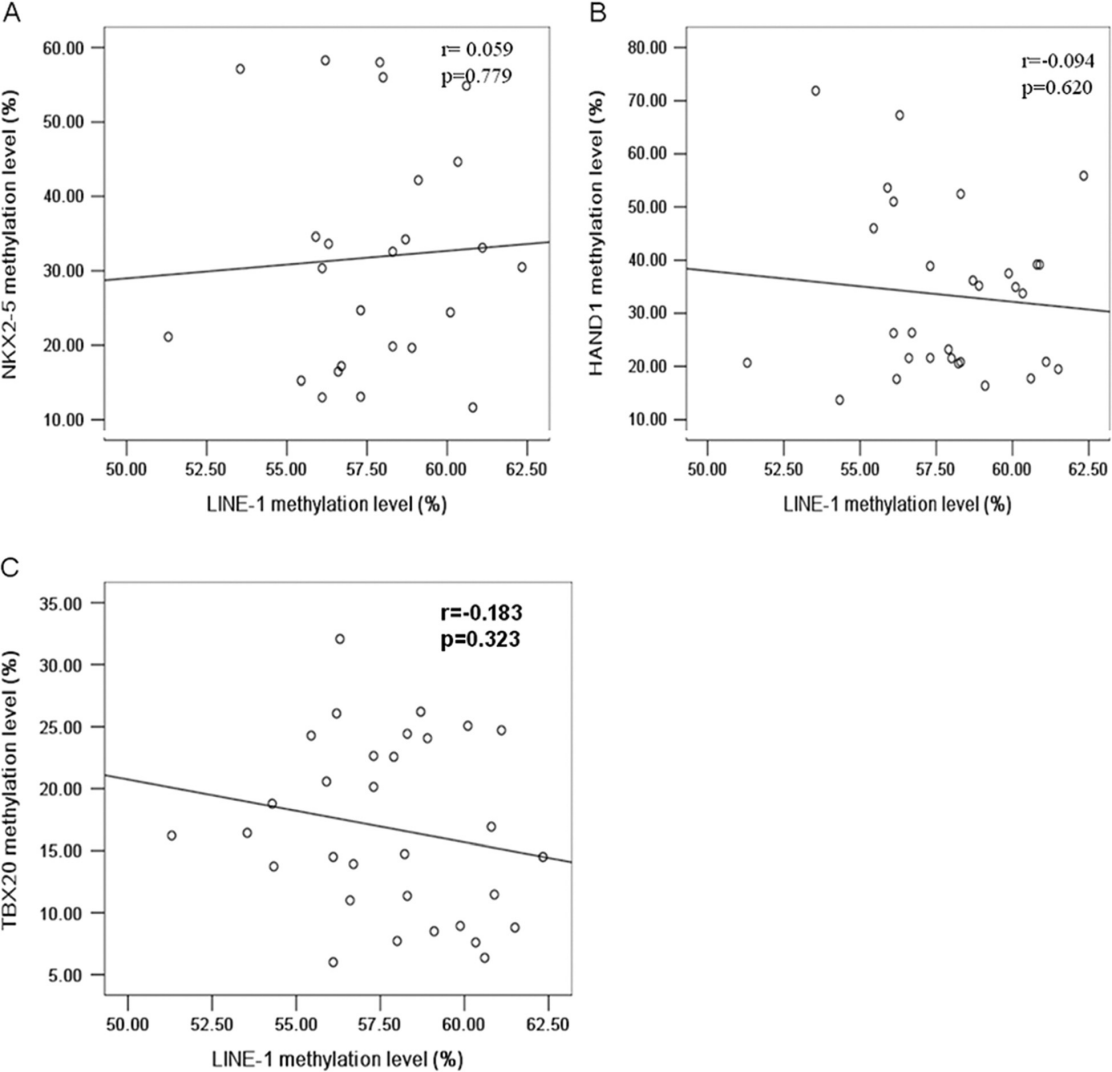
**Correlations among methylation levels of LINE-1 and (A)*NKX2-5*(r = 0.059,*P*= 0.779), (B)*HAND1*(r = − 0.094,*P*= 0.620), and (C)*TBX20*(r = − 0.183,*P*= 0.323) in the TOF patients samples.**

Patients with TOF had significantly higher methylation levels than controls for both *NKX2-5* (30.5% *vs.* 20.0%, *P* = 0.018) and *HAND1* (30.5% *vs.*18.7%, *P* = 0.0006). Lower methylation levels of *TBX20* were observed found in the TOF patients than in controls (16.2% *vs.* 29.5%, *P* < 0.0001; Table 4). No association was found between the methylation levels of *NKX*2-5, *HAND*1 and *TBX*20 and the LINE-1 methylation level (*P* > 0.05; Figure 7).

**Table 4:** Median methylation levels of NKX2-5, HAND1, and TBX20 in the TOF patients and controls

Gene	TOF (median, IQR^1^)	Control (median, IQR)	*P*^2^
*HAND1*	30.5%, 20.8%–40.9%, n = 30	18.7%, 12.2%–24.8%, n =15	0.0006
*TBX20*	16.2%, 11.0%–24.1%, n =31	29.5%, 25.0%–38.9%, n =13	<0.0001
*NKX2-5*	30.5%, 18.4%–43.4%, n =25	20.0%, 13.6%–23.2%, n =13	0.018

^1^*IQR*: interquartile range; ^2^Mann-Whitney U test was used.

## Discussion

LINE-1 methylation patterns can serve as an indicator of global DNA methylation, especially in cancer cell lines [24]. LINE-1 hypomethylation may have two effects on the multistep process of carcinogenesis: facilitating chromosomal instability and controlling gene expression [22]. However, normal tissues from different organs showed tissue-specific levels of methylated LINE-1 [32]. In the present study, we demonstrated that the hypomethylation levels of LINE-1 were present in the cardiac tissue of TOF and might increase the risk of developing TOF. ROC curve analysis confirmed the discriminatory accuracy in predicting the presence of TOF by LINE-1 methylation level, suggesting that hypomethylation of LINE-1 applicable to the risk assessment of TOF. This provides initial evidence of the potential pathophysiology of TOF in pediatric patients. Consistent with one previously published study on the correlation between LINE-1 CpGs[33], the methylation status of the CpG dinucleotides at the LINE-1 promoter regions observed in this study is not equally distributed. The significant hypomethylation observed at CpG units may imply an increase in retro-transposition, which may in turn decrease the chromosomal stability of TOF subjects during early embryonic development. The precise roles of these factors in the development of chromosomal instability and retro-transposition require further study.

Changes in epigenetic patterns from one generation to the next must be evaluated cautiously. These markers are both cell- and tissue-specific and malleable [34]. Many factors, including age, gender and environmental factors, have been shown to influence DNA methylation patterns [31]. Jintaridth et. al studied the relationship between LINE-1 methylation levels and age and found that LINE-1 methylation status was not associated with age in human peripheral blood mononuclear cells [35]. However, age-dependent global DNA demethylation has also been shown to be associated with many diseases, such as gastrointestinal cancer [36]. In families with a history of testicular cancer, researchers have observed strong gender-specific LINE-1 methylation patterns between parents and offspring, particularly between affected fathers and sons [34]. In the present study, we found no association between LINE-1 methylation and either age or gender, suggesting that age and gender may not influence the LINE-1 methylation level. However, we also found significant association between the LINE-1 methylation status and TOF was present in the male group but not in the female group. This is consistent with observations that TOF occurs slightly more often in men than in women [37].

Genome-wide hypomethylation and hypermethylation at promoter CpG islands of specific gene are common in cancer. Recent studies have shown that genome-wide hypomethylation is tightly linked to CpG island hypermethylation in prostate cancer [38] and neuroendocrine tumors [39]. However, global genome hypomethylation has not been found to be associated with promoter hypermethylation for specific genes, as assessed in follicular thyroid cancer [40]. Three genes that regulate heart development, *NKX*2-5, *HAND*1 and *TBX*20, play important roles in the maintenance of normal cardiac development. Although mutations of *NKX*2-5, *HAND*1 and *TBX*20 have been found in patients with TOF, they are present only in a small percentage of patients with congenital heart disease [4]. Based on these findings, we hypothesized that the epigenetic factors related to these genes, such as DNA methylation, are likely to contribute to the development of TOF. In the present study, we found that TOF patients had significantly higher methylation levels in the promoter CpG islands of *NKX*2-5 and *HAND*1 than controls. They had lower methylation levels in the promoter CpG island of *TBX20* (Table 4). These changes were consistent with the reverse results of mRNA expression mircoarray analysis [41]. However, the question of whether or not these mRNA-level changes were caused by the altered methylation status of *NKX*2-5, *HAND*1 and *TBX*20 requires further study. We found no correlation between the methylation status of LINE-1 and that of *NKX*2-5, *HAND*1 and *TBX*20, indicating that the hypomethylation of LINE-1 DNA may not influence the methylation pattern of specific genes in patients with TOF.

## Conclusions

In summary, our results suggest that hypomethylation of LINE-1 may be associated with increased risk of TOF. Aberrant methylation of specific genes was observed in patients with TOF and showed no correlation with the hypomethylation of LINE-1 DNA. Changes in LINE-1 methylation may be useful epigenetic features for TOF patients. The difficulty of collecting heart tissue samples has placed some limitations on this study; we were unable to obtain enough complete matched samples from TOF patients and healthy controls. Further studies with larger sample populations are warranted to confirm our findings.

## Competing interests

The authors declare that they have no competing interests.

## Authors’ contributions

SW participated in study concept and design and coordination perform of the study, helped with the statistical analysis and drafted the manuscript. WH participated in study concept and design and helped to draft the manuscript. QY, WY, MX, and ZF participated in TOF sample acquisition and helped to draft the manuscript. CL and ZP participated in normal control sample acquisition and helped to draft the manuscript. MD and HG participated in study concept and design, study coordination, and helped to draft the manuscript. All authors have read and approved the final manuscript.

## Pre-publication history

The pre-publication history for this paper can be accessed here:

http://www.biomedcentral.com/1755-8794/5/20/prepub
